# Global transcriptome landscape of the rabbit protozoan parasite *Eimeria stiedae*

**DOI:** 10.1186/s13071-021-04811-5

**Published:** 2021-06-07

**Authors:** Yue Xie, Jie Xiao, Xuan Zhou, Xiaobin Gu, Ran He, Jing Xu, Bo Jing, Xuerong Peng, Guangyou Yang

**Affiliations:** 1 Department of Parasitology, College of Veterinary Medicine, Sichuan Agricultural University, Chengdu, 611130 Sichuan China; 2 Institute of Animal Genetics and Breeding, College of Animal Science and Technology, Sichuan Agricultural University, Chengdu, 611130 Sichuan China; 3 Department of Chemistry, College of Life and Basic Science, Sichuan Agricultural University, Chengdu, 611130 Sichuan China

**Keywords:** Coccidiosis, *Eimeria stiedae*, Dynamic development, Parasite–host interactions

## Abstract

**Background:**

Coccidiosis caused by *Eimeria stiedae* is a widespread and economically significant disease of rabbits. The lack of studies on the life-cycle development and host interactions of *E. stiedae* at the molecular level has hampered our understanding of its pathogenesis.

**Methods:**

In this study, we present a comprehensive transcriptome landscape of *E. stiedae* to illustrate its dynamic development from unsporulated oocysts to sporulated oocysts, merozoites, and gametocytes, and to identify genes related to parasite-host interactions during parasitism using combined PacBio single-molecule real-time and Illumina RNA sequencing followed by bioinformatics analysis and qRT-PCR validation.

**Results:**

In total, 12,582 non-redundant full-length transcripts were generated with an average length of 1808 bp from the life-cycle stages of *E. stiedae*. Pairwise comparisons between stages revealed 8775 differentially expressed genes (DEGs) showing highly significant description changes, which compiled a snapshot of the mechanisms underlining asexual and sexual biology of *E. stiedae* including oocyst sporulation between unsporulated and sporulated oocysts; merozoite replication between sporulated oocysts and merozoites; and gametophyte development and gamete generation between merozoites and gametocytes. Further, 248 DEGs were grouped into nine series clusters and five groups by expression patterns, and showed that parasite–host interaction-related genes predominated in merozoites and gametocytes and were mostly involved in steroid biosynthesis and lipid metabolism and carboxylic acid. Additionally, co-expression analyses identified genes associated with development and host invasion in unsporulated and sporulated oocysts and immune interactions during gametocyte parasitism.

**Conclusions:**

This is the first study, to our knowledge, to use the global transcriptome profiles to decipher molecular changes across the *E. stiedae* life cycle, and these results not only provide important information for the molecular characterization of *E. stiedae*, but also offer valuable resources to study other apicomplexan parasites with veterinary and public significance.

**Graphic Abstract:**

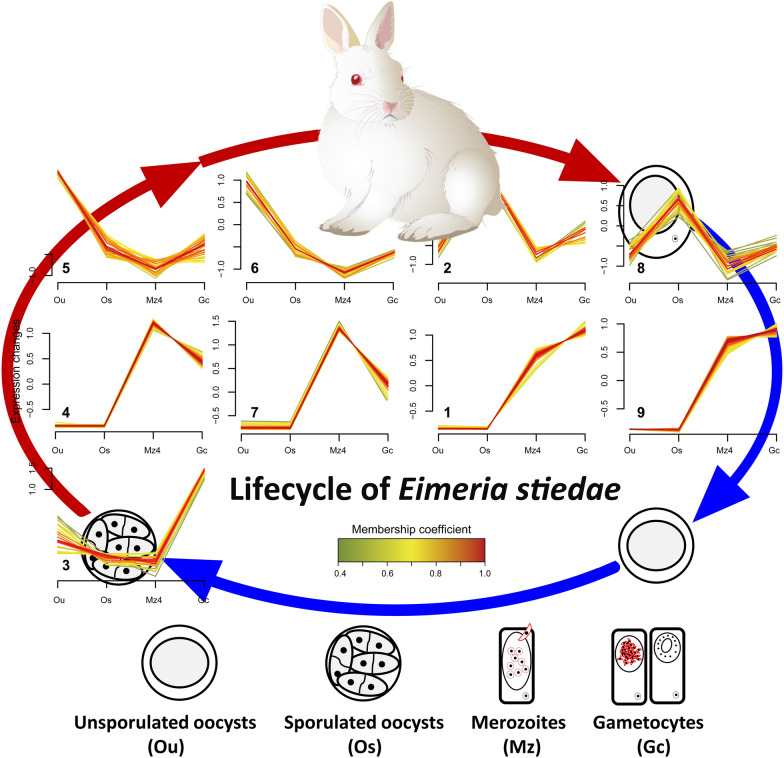

**Supplementary Information:**

The online version contains supplementary material available at 10.1186/s13071-021-04811-5.

## Background

**Fig. 1:**
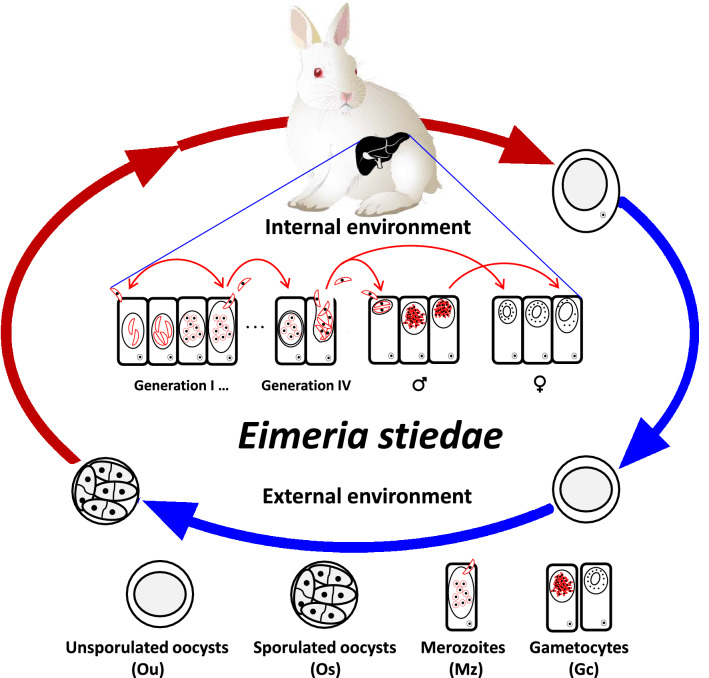
The life cycle of *Eimeria stiedae*. The *E. stiedae* life cycle occurs both in the external environment and within the host rabbit. The oocysts are released in early, unsporulated form (Ou) by hosts in the surroundings. The oocyst undergoes sporogony by meiotic division into four sporoblasts, each of which further develops into one sporocyst. The sporulated oocysts (Os) are then consumed by rabbits, and the released sporozoites penetrate epithelial cells of the small intestine and reach the liver, where they invade liver and bile duct epithelial cells and undergo merogony. The spindle-shaped merozoites (Mz) are set free into the new epithelial cells, in which each grows similar to its parent. In a few days, fissions occur in which the young grow into two types of adults, unlike their parents, comprising male and female gametocytes (Gc). The gametocytes leave the host cell and unite (fertilization) while free in the liver. This procedure is the gametic reproduction (also known as gametogony) and results in production of new unsporulated oocysts, which will be released by the animal in the form of feces, and the life cycle begins again. The entire cycle lasts about 7 days

Rabbit coccidiosis, caused by protozoan parasites of the apicomplexan genus *Eimeria*, is one of the most common and harmful parasitic diseases, leading to huge economic losses in the rabbit industry each year [1–4]. Rabbits of all ages can be infected, especially the young populations between 1 and 4 months of age, with a morbidity and mortality of 90% and 60%, respectively [5–9]. Field investigations show that the coccidia infection rate of rabbits is up to 100%, with significant mixed infections [2, 4, 10]. In China, rabbit coccidiosis has been classified as a level II animal disease by the Ministry of Agriculture because of its high prevalence and resultant economical losses [10, 11]. Currently, 15 species of the genus *Eimeria* are believed to be responsible for rabbit coccidiosis, including *Eimeria stiedae*, *Eimeria magna*, *Eimeria matsubayashi*, *Eimeria neoleporis*, *Eimeria nagpurensis*, *Eimeria irresidua*, *Eimeria flavescens*, *Eimeria piriformis*, *Eimeria intestinalis*, *Eimeria exigua*, *Eimeria elongate*, *Eimeria perforans*, *Eimeria vejdovskyi*, *Eimeria coecicola*, and *Eimeria media* [3, 4, 7, 12, 13]. *E. stiedae* is by far the most pathogenic *Eimeria* species and mainly parasitizes the liver and bile duct epithelial cells of rabbits, resulting in liver coccidiosis [10, 14, 15]. *E. stiedae* has an oral-fecal life cycle and contains three phases [16]: merogony (also known as schizogony), gametogony, and sporogony (Fig. 1). Following the digestion of sporulated oocysts, the sporozoites are released and mainly invade the liver and bile duct epithelial cells, where the merogony phase occurs. Merozoites multiply within and exit from epithelial cells to repeat the process of development through the trophozoite and merogonous stages, and then the life cycle enters the gametogony phase, which produces a new generation of oocysts that are passed out in the feces. Oocyst sporulation (sporogony phase) occurs in the external environment and results in the formation of a new generation of infective oocysts for reinfection. It is clear that during infection, schizogony and gametogony can cause hepatic lesions, and the extent of damage is related to the number of infective oocysts ingested, that leads to a variety of clinical symptoms, such as diarrhea, growth retardation, weight loss, and even death [12, 13].

Similar to other eimerian parasites, *E. stiedae* invades host cells quickly in a highly regulated, parasite-driven manner and needs numerous parasite-derived molecules to achieve subsequent development and induction or modulation of host immune responses [17]. Following an initial interaction with the host cell surface, the parasite synthesizes specialized molecular complexes at the parasite–host interface, which are essential for gliding movement (using the actinomyosin-dependent glideosome) and formation of an invasion “moving junction” (MJ) structure. It has been proven that among the complexes there is a direct correlation between Apple domain-containing microneme proteins (MICs) and host range and tissue tropism [18, 19]. Another class of proteins, apical membrane antigens (AMAs) secreted by the micronemes, can collaborate with secreted rhoptry neck proteins (RONs) to initiate formation of the MJ, against which *Eimeria* can generate a force that allows forward movement into the parasitophorous vacuole [20, 21]. In addition, the glycosylphosphatidylinositol (GPI)-anchored proteins (also known as surface antigens, SAGs) are also found on surfaces of in vivo sporozoites and merozoites of *E. stiedae* (Yuejue Luo, unpublished data). Previous studies showed that *E. tenella* SAGs could bind to various cultured cells, including those from its hosts [1], and their surface locations suggested that SAGs have the ability to suppress cell-mediated immunity and contribute to the marked pro-inflammatory responses and associated pathology during *E. tenella* infection [1, 22]. Unfortunately, little information is available about these homologous molecules related to host cell invasion in *E. stiedae*. Exploration of the evasion strategies that reinforce avoidance of the host immune system, allowing eimerian parasites to survive for years despite strong host immune responses, would be of particular interest to researchers seeking to develop effective interventions. The recent sequencing of the genomes of chicken *Eimeria* and mouse *Eimeria* have provided a better understanding of their pathogenic mechanisms and identified targets to develop control tools leading to new preventions and treatments of coccidiosis. However, we lack omic information about rabbit *Eimeria*. Therefore, the present study aimed to present a high-quality, comprehensive developmental transcriptome analysis of *E. stiedae* and to uncover its interaction with hosts during parasitism using a combination of the PacBio Sequel and the Illumina HiSeq X Ten sequencing platforms. Our study not only provides insights into the developmental biology and pathogenesis of *E. stiedae*, but also produces a set of potentially novel drug and vaccine targets, which could contribute to the development of much-needed new interventions against coccidiosis in rabbits.

## Methods

### Animals and parasite material preparation

Twelve newborn, specific-pathogen-free New Zealand rabbits (*Oryctolagus cuniculus*) were reared in a coccidia-free isolation facility and allowed unlimited access to water and food that were free of any anti-coccidial drugs or antibiotics. To confirm that each rabbit was free of infection before experimental inoculation, their feces were analyzed by salt flotation and light microscopy to ensure the absence of oocysts [23]. All rabbits at 3 weeks of age were infected orally with 2.0 × 10^4^ sporulated oocysts of *E. stiedae.* The Sichuan strain of *E. stiedae*, originally isolated from naturally infected rabbits by the Department of Parasitology, College of Veterinary of Sichuan Agricultural University, was used in this study. We collected four distinct developmental stages of this parasite: unsporulated oocysts (Ou), sporulated oocysts (Os), fourth-generation merozoites (Mz4), and gametocytes (Gc). Specifically, *E. stiedae* Ou were collected from the bile duct epithelial cells at 14 days post-infection (p.i.) and purified through chemical degradation of the tissue with sodium hypochlorite, as described previously [23]. Then, some of the oocysts were continuously sporulated by incubation in a 2.5% (w/v) potassium dichromate solution for 72 h at 28 °C under forced aeration and assessed by microscopic visualization (Os). Mz4 (12 days p.i.) and Gc (14 days p.i.) of *E. stiedae* together with liver tissues (including bile ducts) of rabbits were collected separately with three sample replicates for each stage and stored in liquid nitrogen without host tissue removal because these host RNAs can be easily filtered out using the *O. cuniculus* reference genome [24] (for sample details, please see Additional file 1: Table S1).

### Isolation of total RNA

Before the isolation of total RNA, the oocyst samples including Ou (~ 2 × 10^8^) and Os (~ 1.5 × 10^8^) were cleaned with sodium hypochlorite solution (10–12% active chlorine) for 10 min at 4 °C and resuspended in 0.5 mL deionized water. Oocyst shells were disrupted by vortexing with glass beads (0.5 mm; Sigmund Lindner, Warmensteinach, Germany), followed by incubation at 41 °C in excystation medium: 0.25% (w/v) trypsin (Merck, Darmstadt, Germany) and 1% (w/v) taurodeoxycholic acid (Sigma, St. Louis, MO, USA) in Hanks' balanced salt solution (Sigma), pH 7.4. Then, RNA extraction was prepared by washing each sample (including Ou, Os, Mz4, and Gc) in ice-cold phosphate-buffered saline (PBS) before being mechanically homogenized in Trizol (Invitrogen, Carlsbad, CA, USA) and extracted with 24:1 chloroform/isoamyl alcohol. The aqueous phase was separated by centrifugation and mixed with 0.5 volumes of isopropanol and 4 µL of glycogen (5 mg/mL). Total RNA was precipitated at −80 °C for 1 h, pelleted, and washed with fresh 75% v/v ethanol. The final RNA was resuspended in nuclease-free water and treated with 1 U of DNAse RQI RNAse-free (Promega Corporation, Madison, WI, USA) per 10 µL according to instructions of the manufacturer. The quality and quantity of RNA were determined using a Nanodrop micro-spectrophotometer (Thermo Scientific, Waltham, MA, USA) and an Agilent 2100 bioanalyzer (Agilent Technologies, Santa Clara, CA, USA).

### PacBio SMRT sequencing library preparation and sequencing

The isolated RNAs from the Ou, Os, Mz4, and Gc samples were pooled in equal amounts to provide the total RNA for PacBio SMRT sequencing. The mRNA was isolated from the total RNA using magnetic oligo-dT beads and reversely transcribed into cDNA using a Clontech SMARTer PCR cDNA Synthesis Kit (Clontech Laboratories, Inc. CA, USA). Size selection of the PCR products was performed using the BluePippin™ Size Selection System (Sage Science, Beverly, MA, USA), and the fragments ranging from 0.5 to 6 kb were retained. Afterwards, large-scale polymerase chain reaction (PCR) was carried out to amplify the full-length cDNA. The ends of the cDNAs were repaired before the sequencing adapters were ligated to the cDNAs. SMRT-bell template libraries were constructed from the obtained cDNAs and sequenced on the PacBio Sequel platform.

### Illumina RNA-Seq library preparation and sequencing

RNA samples from Ou, Os, Mz4, and Gc samples were used for Illumina (San Diego, CA, USA) library construction and sequencing, respectively. The cDNA libraries for Illumina HiSeq X Ten sequencing were constructed as follows: mRNA was enriched from the total RNA using the magnetic oligo-dT bead binding method and sheared into short fragments using fragmentation buffer. Then, the short mRNAs were used as templates to synthesize double-stranded cDNAs using random primers by reverse transcription. The cDNA fragments were purified using a QiaQuick PCR extraction kit (Qiagen, Venlo, Netherlands) and ligated with Illumina sequencing adapters. The ligation products were size-selected by agarose gel electrophoresis and enriched by PCR to construct the cDNA libraries, which were sequenced on the Illumina HiSeq X Ten platform. All the sequencing works were carried out at Wuhan Frasergen Bioinformatics Co., Ltd. (Wuhan, China). After RNA sequencing (RNA-Seq), the raw reads were filtered by removing adaptors, reads containing more than 10% of unknown nucleotides, and low-quality reads. The Q20/Q30 and GC content of the clean reads were calculated.

### Analysis of the Iso-Seq data

The SMRT analysis pipeline v5.0.1 was used to process raw sequencing data from the PacBio SMRT sequencing. Sub-reads were screened and subjected to generation of high-quality circular consensus sequence (CCS) reads. The CCS reads were further classified into full-length non-chimeric (FLNC) reads, full-length chimeric reads, non-full-length reads, and short reads according to whether the 5′ primer-adapters, 3′ primer-adapters, and polyA tail signal were simultaneously observed. The short reads were discarded, and the FLNC reads were clustered using the tool of iterative clustering and error correction (ICEC) to generate the cluster consensus isoforms. To improve the accuracy of the full-length transcripts, we first used the non-full-length reads to polish the obtained cluster consensus isoforms, followed by using Quiver to obtain the full-length, high-quality consensus sequences (accuracy ≥ 99%). Additionally, the low-quality isoforms were also corrected using filtered Illumina RNA-Seq reads by the Long-Read De Bruijn Graph Error Correction (LoRDEC) tool, as previously described [25]. All of these isoform sequences were finally filtered using CD-HIT v4.6.7 (https://github.com/weizhongli/cdhit/releases) to remove redundant sequences, with a threshold of 99% identity, and compared with the rabbit reference genome [24] to separate *E. stiedae* full-length transcriptomes.

For comprehensive functional annotations, the full-length transcripts of *E. stiedae* were searched against public protein databases, including the National Center for Biotechnology Information (NCBI) non-redundant protein (Nr) (https://www.ncbi.nlm.nih.gov/), Swiss-Prot (https://www.expasy.ch/sprot/), Cluster of Orthologous Groups of proteins (COG/KOG) (https://www.ncbi.nlm.nih.gov/COG), and Kyoto Encyclopedia of Genes and Genomes (KEGG) (https://www.genome.jp/kegg/) databases using the BLASTX program (https://www.ncbi.nlm.nih.gov/BLAST/) with a cutoff E-value ≤ 10^−5^. Gene Ontology (GO) annotation was performed using the Blast2GO software [26], and GO categories were retrieved using the WEGO software [27]. Moreover, comparisons of transcript similarity were also performed using the protozoan ToxDB databases (https://toxodb.org/toxo/).

### Differential expression analysis using the RNA-Seq data

Given that the Mz4 and Gc samples contained both parasite and host RNAs, clean paired-end RNA-Seq reads from the Mz4 and Gc libraries were mapped to the rabbit reference genome [24] using TopHat v.2 [28] to remove the rabbit sequences. Then, the remaining reads, together with those from the Ou and Os libraries, were aligned against the full-length transcripts of *E. stiedae* yielded from SMRT sequencing. The resulting alignments were used to estimate gene abundances. The gene expression levels in the four stages of the parasite were normalized by FPKM (fragments per kilobase per million mapped reads) using bowtie2 included in RSEM [29]. Raw and normalized mapped reads per gene and sample replicates are given in Additional file 2: Table S2 and Additional file 3: Table S3, respectively. Differentially expressed genes (DEGs) were determined using DESeq2 [30] based on pairwise comparisons between Ou, Os, Mz4, and Gc of *E. stiedae*. A false discovery rate (FDR) < 0.05 and a fold change ≥ 1 were used as the thresholds to identify DEGs. Series-cluster analysis was used specifically in the four stages of *E. stiedae* for DEGs classification according to the FPKM values of the genes, and multiple comparison test and Fisher’s exact test were selected to calculate the significance levels of the profiles [31, 32].

In addition, gene co-expression network analysis was carried out to track the genes closely related to the life-cycle stages of *E. stiedae*, especially to its infective sporulated oocyst stage and parasitic merozoite and gametocyte stages. For this purpose, the reads per kilobase of transcript per million mapped reads (RPKM) values of all transcripts were used to establish this co-expression network using the weighted gene co-expression network analysis (WGCNA) implemented in the R package (https://horvath.genetics.ucla.edu/html/CoexpressionNetwork/Rpackages/WGCNA/index.html). To construct an adjacency matrix, the power adjacency function was adopted with a soft thresholding power of 14, which resulted in a scale-free topology fit index of 0.89. Then, the adjacency matrix was converted to a topological overlap matrix (TOM), and the dissimilarity TOM (1 − TOM) was calculated [33]. Modules were selected using the dynamic tree cut algorithm. Correlation between the modules and stages was calculated based on the Pearson correlation coefficient between the expression profiles of each module along the stages, as suggested in the WGCNA tutorial (https://horvath.genetics.ucla.edu/html/CoexpressionNetwork/Rpackages/WGCNA/Tutorials/). The association of an individual gene with a trait was quantified using gene significance (GS) values, and the trait in our case was always the stage of higher absolute Pearson correlation coefficient with the module that the transcript belonged to. Specifically, if a transcript that belongs to the blue module (most highly correlated with the Os stage, see Results), the correlation was calculated between the expression of this transcript in Os and the expression of the same transcript in all the other three stages.

### Validation of RNA-Seq data using qRT-PCR

A total of 60 DEGs identified by the above RNA-Seq analysis, across each pairwise comparison between Ou, Os, Mz4, or Gc (ten per pair), were selected for validation by quantitative reverse transcriptase PCR (qRT-PCR). cDNAs were synthesized by reverse transcription from the same total RNA that was used for the RNA-Seq experiments at the corresponding stage using a RevertAid First Strand cDNA Synthesis Kit (Thermo Fisher Scientific, Vilnius, Lithuania) according to the manufacturer’s instructions. The cDNAs were then used as templates for the qPCR reactions, carried out in a MX300P spectrofluorometric thermal cycler (Stratagene, La Jolla, CA, USA) three times independently, using the housekeeping gene *GAPDH* (glyceraldehyde-3-phosphate dehydrogenase) as a control. The qPCR primers are described in Additional file 4: Table S4. The qPCR cycling conditions were as follows: 95 °C for 5 min, followed by 40 cycles of 95 °C for 10 s, 59 °C for 10 s, and 72 °C for 15 s. The temperatures of the melt curve analysis ranged from 72 to 95 °C to ensure the specificity of qPCR products. The relative gene expression was calculated using the 2^−△△CT^ method.

## Results

### De novo transcriptome assemblies

**Fig. 2:**
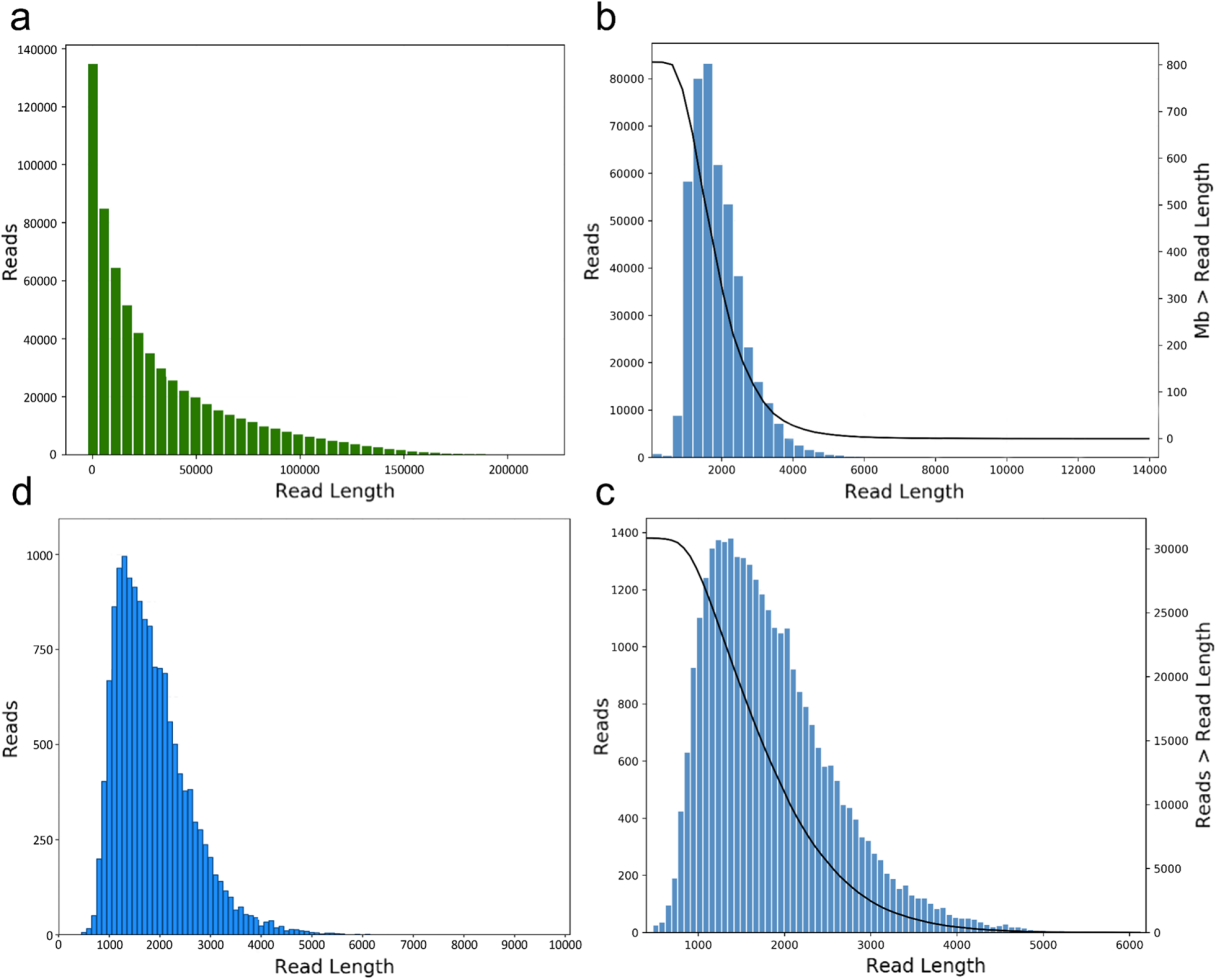
Summary of PacBio SMRT sequencing. **a** The number and length distributions of PacBio 21,313,777,289 reads. **b** The number and length distributions of 456,652 circular consensus sequence (CCS) reads. **c** The number and length distributions of 14,892 high-quality (HQ) transcripts. **d** The number and length distributions of 12,582 non-redundant transcript isoforms of *Eimeria stiedae*

The short-read RNA-Seq and long-read PacBio Iso-Seq were combined to characterize the *E. stiedae* transcriptome. Specifically, 12 RNA samples from four developmental stages (Ou, Os, Mz4, and Gc) of *E. stiedae* were sequenced on the Illumina HiSeq X Ten platform. After quality filtering, a total of 976 million 150-bp long reads were generated (Table 1). To obtain a wide coverage of *E. stiedae* transcriptome, a pooled sample representing high-quality RNA from the aforementioned four stages (Ou, Os, Mz4, and Gc) was sequenced using the PacBio RS II platform. A total of 21.3 Gb of raw reads were generated, and 20,769,372,093 sub-reads were obtained after filtering. Subsequently, the SMRT pipeline was used to process the raw sequencing data. In total, 456,652 CCS reads were obtained and included 385,471 FLNC reads (Fig. 2; Additional file 5: Table S5). To solve the high error rate and improve the accuracy of the PacBio reads, the ICEC tool in the SMRT Link and Quiver programs was used for CCS sequence clustering. Then, 14,892 polished high-quality (HQ) transcripts were obtained. After error correction by the RNA-Seq data of four different developmental stages (Ou, Os, Mz4, and Gc) of *E. stiedae* and clearance of the redundant sequences using the CD-Hit program, 12,582 non-redundant transcript isoforms of *E. stiedae* were generated (Fig. 2; Table 2). The length of transcripts ranged from 492 to 6009 bp with an average length of 1808 bp and an N50 of 1972 bp. Further, the BUSCO (v3.0) software was used to benchmark the transcriptome completeness by assessing essential single-copy orthologs (*n* = 502) of the coccidia lineage. The results showed that the completeness of the assembly was up to 97.21%, with only 1.00% of transcripts missing (Additional file 6: Table S6). Thus, the *E. stiedae* transcriptome was capable of providing a solid data basis for subsequent analyses.

**Table 1:** Summary of the illumina data

Sample	Clean reads	Clean bases (bp)	Q20 (%)	Q30 (%)	GC (%)	Mapped reads (%)
Ou1	60,769,406	9,115,410,900	97.2	92.5	57.0	63.81
Ou2	49,275,606	7,391,340,900	95.9	89.7	57.1	54.58
Ou3	58,339,712	8,750,956,800	97.2	92.7	57.1	59.67
Os1	56,474,446	8,471,166,900	97.8	93.7	56.2	67.82
Os2	53,036,154	7,955,423,100	98.3	94.9	56.1	67.71
Os3	52,274,774	7,841,216,100	98.0	94.2	56.1	67.30
Mz41	115,921,974	17,388,296,100	97.2	92.3	52.8	63.00
Mz42	104,582,964	15,687,444,600	97.2	92.5	53.8	66.05
Mz43	98,733,052	14,809,957,800	96.0	90.1	53.3	65.82
Gc1	115,143,520	17,271,528,000	97.2	92.6	55.3	66.60
Gc2	105,156,874	15,773,531,100	97.0	92.1	55.7	66.85
Gc3	106,508,822	15,976,323,300	96.4	90.7	54.4	66.86

Triple duplication per stage

Ou: unsporulated oocysts; Os: sporulated oocysts; MZ4: fourth-generation merozoites; Gz: gametocytes

**Table 2:** Statistics of the non-redundant reads

Total number	Total bases	Average length (bp)	Maximum length (bp)	Minimum length (bp)	Median length (bp)	N50 (bp)
12,582	26,912,648	1808	6009	492	1651	1972

In addition, our BLASTx similarity analysis against the protozoan ToxoDB database demonstrated that the *E. stiedae* full-length transcripts were similar to those of several *Eimeria* species (Additional file 7: Figure S1). Among them, 4156 (33.03%) transcripts showed significant homology with those of *Eimeria falciformis*, followed by 1498 (11.91%), 1289 (10.25%), 987 (7.84%), 843 (6.70%), 814 (6.47%), 705 (5.61%), and 432 (3.43%) transcripts similar to sequences from *Eimeria tenella*, *Eimeria necatrix*, *Eimeria acervulina*, *Eimeria mitis*, *Eimeria brunetti*, *Eimeria praecox*, and *Eimeria maxima*, respectively.

### Function annotation of the full-length *E. stiedae* transcriptome

**Fig. 3:**
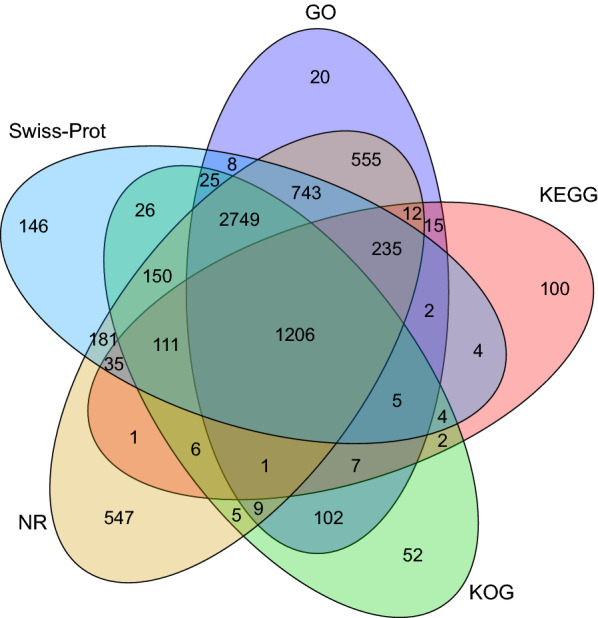
Annotation of the full-length transcripts of *Eimeria stiedae*

To obtain a comprehensive annotation of the *E. stiedae* transcriptome, 12,582 full-length transcripts were annotated by searching against public protein databases. In total, 6546, 5630, 4460, 1746, and 5694 transcripts were separately annotated in the Nr, Swiss-Prot, KOG, KEGG, and GO databases, respectively, and 1206 transcripts were annotated in all five databases (Fig. 3; Additional file 8: Table S7). In addition, 5538 unannotated transcripts might represent novel *E. stiedae* genes, because no homologs were detected in any database.

**Fig. 4:**
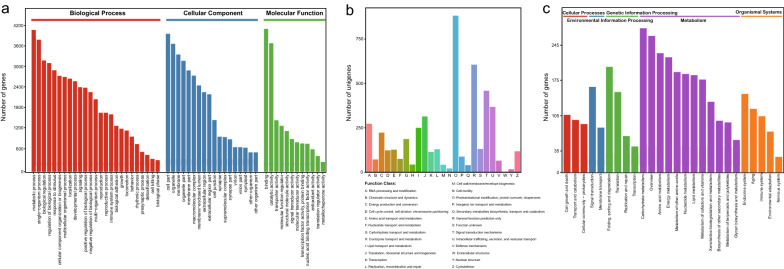
Function annotation and classification of *Eimeria stiedae* full-length transcripts. **a** Gene Ontology (GO) enrichment analysis of transcripts, including biological process, cellular component, and molecular function. **b** Cluster of orthologous groups (COG) classification analysis of transcripts. **c** Kyoto Encyclopedia of Genes and Genomes (KEGG) enrichment analysis of transcripts, including cellular processes, environmental information processing, metabolism, and organismal systems

GO-based functional enrichment analysis was used to assign the functions of the full-length transcripts to molecular function, cellular component, and biological process terms (Fig. 4a; Additional file 9: Table S8). Notably, the biological process represented the majority of the GO terms. In addition, 1125 and 1978 transcripts were assigned to molecular function and cellular component, respectively. A high proportion of genes were assigned to GO classes such as cellular process, metabolic process, single-organism process, cell part, organelle, binding, and catalytic activity, which are involved in metabolite biosynthesis and are important for parasitism of the protozoan *Eimeria*. The KOG analysis demonstrated that 4460 transcripts were assigned to 26 functional clusters. As shown in Fig. 4b, the five largest categories were “Posttranslational modification, protein turnover and chaperones” (882, 19.78%), “General function prediction only” (606, 13.59%), “Signal transduction mechanisms” (459, 10.29%), “Intracellular trafficking, secretion, and vesicular transport” (368, 8.25%), and “RNA processing and modification” (273, 6.12%). Although fewer genes were annotated by the KEGG analysis compared with the other databases, it can still assist identification of functional genes and is helpful to understand the functions and interactions of genes in biosynthetic pathways [34]. The KEGG analysis assigned 1746 transcripts from *E. stiedae* to five major biological pathways (Fig. 4c; Additional file 10: Table S9). The largest group was metabolic pathways, containing 1426 transcripts. Moreover, many transcripts were assigned to other important pathways, including organismal systems, genetic information processing, cell processes, and microbial metabolism in diverse environments.

### Differentially expressed genes (DEGs) and qRT-PCR validation

**Fig. 5:**
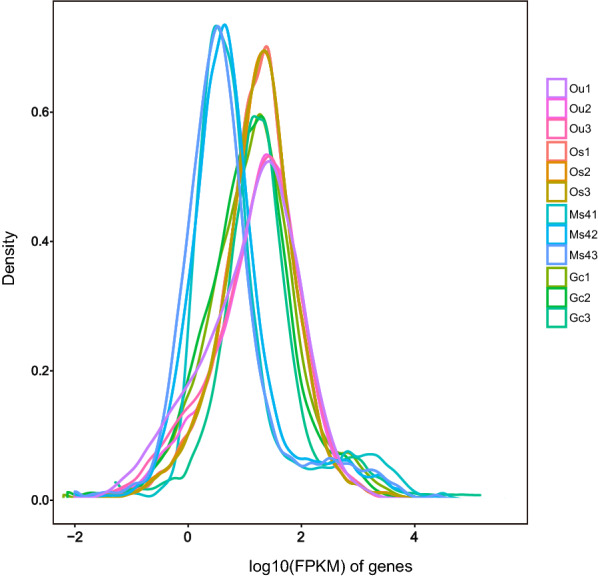
The FPKM distribution of different developmental stages of *Eimeria stiedae*, with triple duplication per stage

**Fig. 6:**
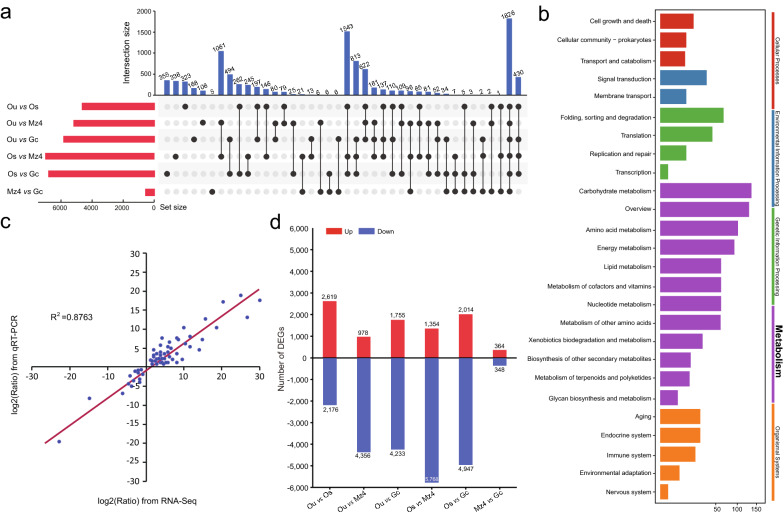
Analysis of differentially expressed transcripts between stages of *Eimeria stiedae*. **a** An UpSet plot showing the sets of differentially expressed transcripts from four different developmental stages, including the quantitative analysis of aggregate intersections between stages. The vertical bars show the number of intersecting transcripts between stages, denoted by the connected black circles below the histogram. The horizontal bars show the transcript set size between stages. **b** A summary of the numbers of up- and downregulated differentially expressed transcripts. **c** Validation of RNA-Seq results by quantitative real-time reverse transcription PCR (qRT-PCR). Plot of gene expression (fold change) determined by the RNA-Seq (X-axis) and qRT-PCR (Y-axis) of 60 selected genes (Pearson’s correlation, *R*^2^ = 0.8763, *p* < 0.01). The fold change of expression was expressed as log2 values. **d** Kyoto Encyclopedia of Genes and Genomes (KEGG) enrichment analysis of differentially expressed transcripts, including cellular processes, environmental information processing, metabolism, and organismal systems

To investigate and understand the variation of transcript abundance and expression patterns of genes among Ou, Os, Mz4, and Gc of *E. stiedae*, the RNA-Seq clean reads were mapped to the SMRT transcripts to determine the expression level with FPKM-normalized read counts. The average of mapped reads was 64.35% (Table 1), and the FPKM distribution of all samples is shown in Fig. 5. After comparative analysis of the data between Ou, Os, Mz4, and Gc, a total of 10,057 DEGs were detected in at least one pairwise comparison (Ou vs. Os, Ou vs. Mz4, Ou vs. Gc, Os vs. Mz4, Os vs. Gc, and Mz4 vs. Gc) (Table 3; Fig. 6a; Additional file 11: Table S10). As shown in Fig. 6b, Os and Mz4 had the most specific DEGs (7122), while Mz4 and Gc had fewer DEGs (712), suggesting larger biological differences between the in vitro oocysts and the parasitizing phase merozoites and fewer differences between in vivo merozoites and gametocytes of *E. stiedae*. Among them, 1354 genes were upregulated, and 5768 genes were downregulated between Os and Mz4, and 364 genes were upregulated and 348 genes were downregulated between Mz4 and Gc. Moreover, comparisons between Ou vs. Os, Ou vs. Mz4, Ou vs. Gc, and Os vs. Gc revealed 4795 (2619 upregulated and 2176 downregulated), 5334 (978 upregulated and 4356 downregulated), 5988 (1755 upregulated and 4233 downregulated), and 6961 (2014 upregulated and 4947 downregulated) DEGs, respectively. We randomly selected 60 DEGs for qRT-PCR quantification from the same RNA samples of Ou, Os, Mz4, and Gc to validate whether our sequencing and analysis were reliable. The high linear correlations and Pearson correlation coefficient between the qRT-PCR and RNA-Seq results supported the validity of our transcriptomic RNA-Seq data and guaranteed reasonable deductions from the gene expression values generated from RNA-Seq (Fig. 6c).

**Table 3:** Number of differentially expressed transcripts between *Eimeria stiedae* stages

Stage comparison	Upregulated transcripts	Downregulated transcripts	Total
Ou vs. Os	2619	2176	4795
Ou vs. Mz4	978	4356	5334
Ou vs. Gc	1755	4233	5988
Os vs. Mz4	1354	5768	7122
Os vs. Gc	2014	4947	6961
Gc vs. Mz4	364	348	712

Triple duplication per stage and *q* value < 0.05

Ou: unsporulated oocysts; Os: sporulated oocysts; MZ4: fourth-generation merozoites; Gz: gametocytes

In addition, 1826 genes were differentially expressed in all comparison groups, suggesting that these genes might play significant roles in the different stages of *E. stiedae*. Furthermore, KEGG functional enrichment analysis of these transcripts revealed a wide range of metabolism classes presented in the four developmental stages of *E. stiedae*, which mainly involved carbohydrate metabolism, energy metabolism, amino acid metabolism, lipid metabolism, and nucleotide metabolism (Fig. 6d).

### Overview of transcriptomic dynamics

**Fig. 7:**
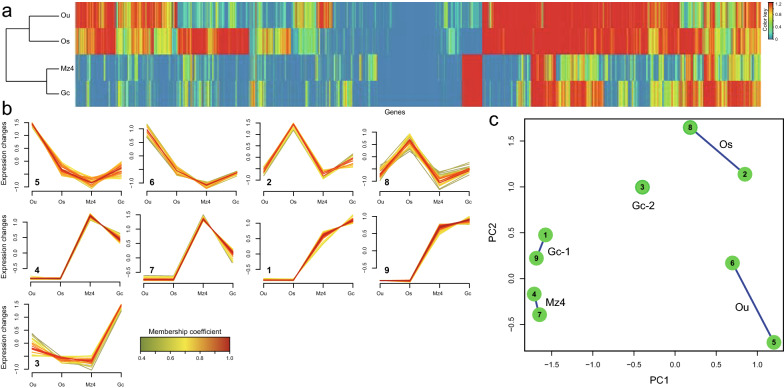
Transcription throughout the development of *Eimeria stiedae*. **a** Heat map showing the transcriptional levels for the genes either specific to a stage or overexpressed in a stage. Based on these, the free-living stages (unsporulated oocysts [Ou] and sporulated oocysts [Os]) cluster separately from the parasitic stages (fourth-generation merozoites [Mz4] and gametocytes [Gc]). **b** Soft clustering of all genes with non-zero FPKM (fragments per kilobase per million mapped reads) in the four stages was included. Nine clusters associated into five different groups based on the extent of shared genes among them, each characterized primarily by having high level of normalized transcription in one of these four developmental stages. **c** Cluster overlap panel for gene clusters presented in (**d**). Genes that did not have > 0.5 membership coefficient in any of the clusters were not assigned to any clusters and are not shown here

Based on the analysis of DEGs across the life cycle (including Ou, Os, Mz, and Gc), we obtained an opportunity to view the gene expression landscapes during *E. stiedae* development. The results revealed the dynamics of transcription across the *E. stiedae* life cycle, with 8775 genes showing highly significant (FDR < 10^−5^) expression changes between different life stages (Fig. 7a; Additional file 12: Figure S2, Additional file 13: Figure S3 and Additional file 14: Table S11). From Ou to Os, the transition was associated with a large-scale activation of transcription of genes encoding a substantial number of organelle-related components, including integral component of membrane, intrinsic component of membrane, rhoptry, pellicle, inner membrane complex, microtubules, and granules, supporting the biological process in which one diploid unsporulated oocyst divides into eight haploid sporozoites and then subcellular organelles appear. Additionally, genes associated with protein kinase activity, channels, and bindings, as well as oxidoreductase activity, were also upregulated and probably reflected the increased chemosensation and/or proprioception to the external environment and the need for detoxification of endogenous waste in the infective Os. Once ingested by the host animal, the transition from the Os to the parasitic Mz and Gc stages saw a renewed and massive surge in the number of DEGs, which were enriched in cell component-linked GO terms, including intracellular organelle, ribosomal subunit, cytoplasm, intracellular ribonucleoprotein complex, and macromolecular complex; and biological process-linked GO terms, including metabolic process, single-organism process, pole cell migration, regulation of biological process, response to stimulus, localization, signaling, and reproductive process. These transcription changes not only linked the transition from the free-living to parasitic stages, but also reflected the host invasion and the following merozoite replication, gametogony, and parasite–host interactions (mainly involving immunoregulation). Indeed, from Mz4 to Gc, we noted that numerous DEGs were significantly enriched (corrected *p* < 0.05) in immune-related GO terms, such as positive regulation of interleukin-2 production, neutrophil homeostasis, and interleukin-2 production. Besides, many DEGs significantly enriched (corrected *p* < 0.05) in protein translation-related GO terms were also observed, such as amide biosynthetic process, peptide biosynthetic process, peptide metabolic process, and cellular amide metabolic process. KEGG enrichment analysis showed that the “Ribosome” pathway was positively related to these protein GO terms, suggesting a more active protein expression in both Mz and Gc stages compared with that in Os. Interestingly, limited expression differences were observed between the Mz and Gc stages, and most transcriptions were associated with gametophyte development, female/male gamete generation, and microgametogenesis, which are specifically related to gametogony (see Fig. 6a, d; Additional file 14: Table S11).

After the overview of transcriptomic dynamics of *E. stiedae*, we further focused on genes that act at the infection (sporogony) and host parasitism (schizogony and gametogony) stages and aimed to provided drug, vaccine, and diagnostic antigen candidates for development of new and urgently needed interventions against rabbit and other animal coccidioses. In this part, we performed further analyses of the DEGs at the four developmental stages (Ou, Os, Mz, and Gc) of *E. stiedae* using series cluster analysis because of their associations with host infection and parasitism during the life cycle. Based on 248 DEGs, a total of nine series clusters were generated (Fig. 7b). These clusters were subsequently divided into five groups according to their gene expression patterns. Attention was paid to groups that uniquely described each stage (Fig. 7c; Additional file 15: Table S12). These groups gathered the genes with similar functions; therefore, they could reflect the main biological events occurring at the targeted stage. Within the group that uniquely described the Ou stage, both clusters 5 and 6 had 27 (10.89%) genes, which declined gradually from Ou to Mz4, and maintained a relative low expression from Mz4 to Gc. GO enrichment analysis showed an expansion of transcription concerned with the sporogony process, including cell proliferation (e.g., PRP19, U2 spliceosome, AP3, cadherin, and eIF2), organelle/organelle membrane (e.g., LROs, LAMP1, lEM, EEM, ER, and TBC), signaling pathway (e.g., IFN-α/β, LGICs, and AKT2), oxidoreductase activity (e.g., BADH, GSTs, and P450s), and carbohydrate metabolism (e.g., GALU, GALT, and UTases) as well as other molecular binding and/or transport (e.g., HSP, SNARE, and PDK). We also noted that autophagy was active in this stage, and its enriched HOPS and CORVET complexes were enriched here and were probably associated with organelle clearance during oocyst differentiation and sporozoite development [35]. Next, the group that only showed an expression peak at the Os stage contained clusters 2 and 8. A total of 16 genes (6.45%) were identified in both clusters. Although most of these genes had no GO terms related to sporogony, the genes encoding glideosome-associated proteins (GAP45 and GAP50), serine/threonine protein kinases (STPKs), and integral components of the membrane provided key information regarding the interactions between Os and host cells during invasion. These glideosome components have been proven to enable the motile stage of coccidian parasites (including sporozoites and merozoites) to move towards potential host cells and enter them by pushing forward the membrane of host cells [35–37]. After the switch to host parasitic stages, there were two groups including five expression clusters that specifically described the Mz4 and Gc stages. In the Mz4 group, clusters 4 and 7 contained 42 (16.94%) genes that maintained a relative stable expression in the Ou and Os, and then increased at Mz4 and declined at Gc. There was a major increase in the number of genes transcribed specifically in such a crucial life-cycle transition from free-living to parasitic phase; the enrichment was related to mitochondrion organization, cell proliferation, exocytosis, steroid/cholesterol/isoprenoid metabolic or biosynthetic process (e.g., HSP70, CL, and MDC), signaling (e.g., Wnt, IκB/NFκB, FGFR, MAPK, and ERBB2), protein ubiquitination (e.g., ERAD, UPD, and E2), and immune response (e.g., APC, CD81, and MHC II molecules), as well as DNA binding and repair (e.g., AP2). Most of these genes were inferred to be associated with extensive asexual replication and immune-related processes of *E. stiedae* as it inhibited the bile duct epithelial cells of the host animal. For the group that uniquely described the Gc stage, compared to cluster 3, clusters 1 and 9 shared a similar expression pattern, in which 36 (14.52%) genes had a relative stable expression from Ou to Os, and increased through Mz4, and reached maximum expression at Gc. Among these genes, there was an abundance of transcripts related to sexual replication, such as mitotic cell cycle, germ cell development, oocyte localization-involved germarium-derived egg chamber, and ovarian follicle cell. Besides, genes related to receptor-mediated virion attachment to the host cell, viral entry into the host cell, and immune-related molecules (e.g., ICOS and AGPAT) as well as protein translation, metabolic process, oxidation–reduction process, and tRNA processing, were enriched in the Gc stage.

### Co-expression analysis

**Fig. 8:**
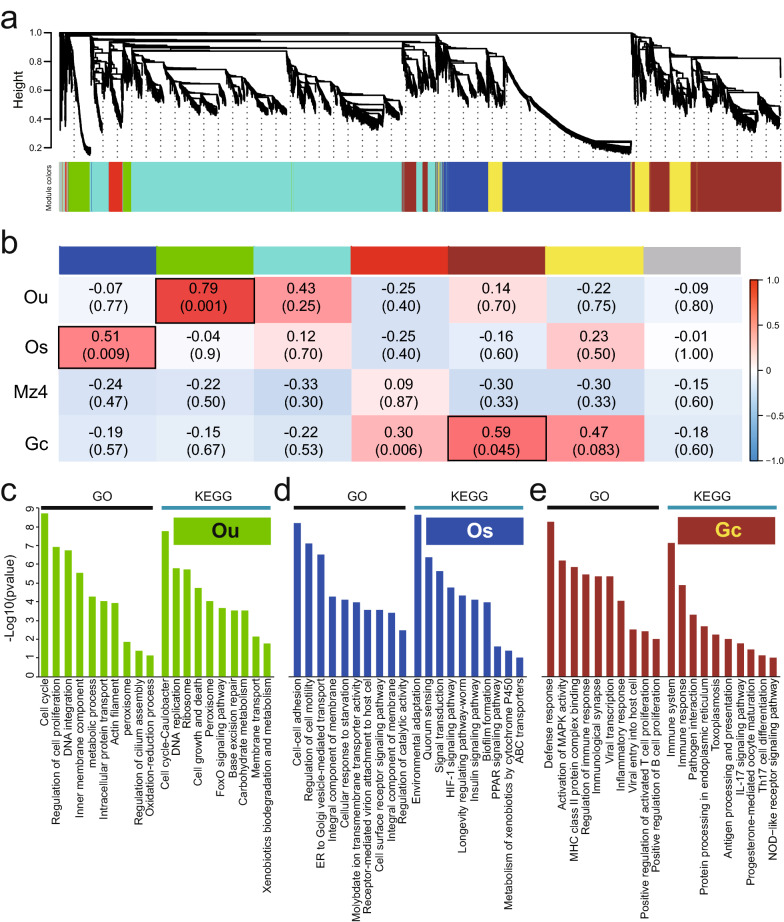
Weighted gene co-expression network analysis (WGCNA) of genes closely related to the life-cycle stages of *Eimeria stiedae*. **a** Gene dendrogram with the corresponding seven modules. Each color represents a module with highly connected genes. **b** Relationships of module eigengenes and developmental stages, including unsporulated oocysts (Ou), sporulated oocysts (Os), fourth-generation merozoites (Mz4), and gametocytes (Gc). The numbers in the table represent the Pearson correlation coefficients between the corresponding module eigengene and trait, with the *p* values in brackets. Furthermore, three upregulated modules including MEgreen, MEblue, and MEbrown were chosen for Gene Ontology (GO) and Kyoto Encyclopedia of Genes and Genomes (KEGG) enrichments because they significantly correlated with Ou (**c**), Os (**d**), and Gc (**e**), respectively. The top 10 GO terms are listed according to the enrichment *p* value

To further explore genes closely related to the life-cycle stages of *E. stiedae*, we carried out a weighted gene co-expression network analysis. The file containing the expression levels of all transcripts from the Ou, Os, Mz, and Gc stages was included in the analysis. Stage samples were correctly clustered in a dendrogram by their expression correlations (Additional file 16: Figure S4). We obtained seven different co-expression modules with sizes ranging from 52 to 3929 transcripts, including the gray module denoting unassigned transcripts (Table 4; Fig. 8a). To visualize the results, we plotted a heatmap to depict the topological overlap matrix among these transcripts (Additional file 17: Figure S5). Next, we estimated the relationship of the module eigengene (ME) to each stage using Pearson correlation analysis. It was obvious that, except for Mz4, each stage contained at least one module whose gene expression had a statistically significant positive correlation with that stage (Fig. 8b; Additional file 18: Figure S6), thus providing us with another opportunity to probe the genes genuinely related to the life-cycle stages of *E. stiedae*, especially the infective and parasitic stages. We focused on three upregulated modules (MEgreen, MEblue, and MEbrown) because they correlated significantly with Ou (*r*^*2*^ = 0.79, *p* = 0.001), Os (*r*^*2*^ = 0.51, *p* = 0.009), and Gc (*r*^*2*^ = 0.59, *p* = 0.045), respectively (see the black boxes in Fig. 8b). Functional enrichment showed that most of the genes in the MEgreen module correlated with the Ou and were significantly enriched with processes related to the sporogony, as suggested by GO terms such as regulation of cell proliferation, inner membrane component, actin filament, and cilium assembly, as well as KEGG pathways such as DNA replication, cell growth and death, ribosome, and FoxO signaling (Fig. 8c). The MEblue module correlated with the Os, and the genes were significantly upregulated and enriched with GO processes associated with host cell invasion and environmental adaptation, such as G cell adhesion and motility, ER to Golgi vesicle-mediated transport, receptor-mediated virion attachment to host cell, and cell surface receptor signaling pathway, and KEGG pathways such as quorum sensing, HIF-1 signaling, and longevity-related pathways (Fig. 8d), once again supporting its role as the main infection source for the host animal. Besides, the genes in MEbrown module were upregulated in parasitic stage Gc and were mostly enriched in host immune-related biological processes (e.g., MHC class II binding, T-cell proliferation, and B-cell proliferation) and pathways (e.g., pathogen interaction, antigen processing, and presentation, IL-17 signaling, and Th17 cell differentiation; Fig. 8e).

**Table 4:** Number of transcripts per module and module-correlated stages of *Eimeria stiedae*

Module color	No. of transcripts	% of transcripts	Stage with a higher absolute correlation value
MEblue	2259	24.59	Os
MEgreen	383	4.17	Ou
MEturquoise	3929	42.77	Ou
MEred	192	2.09	Gc
MEbrown	1675	18.23	Gc
MEyellow	696	7.58	Gc

Ou: unsporulated oocysts; Os: sporulated oocysts; Gz: gametocytes

## Discussion

As an important protozoan parasite, *E. stiedae* poses significant threats to rabbit breeding worldwide [10, 14, 15]. However, until now, the genome and transcriptome of *E. stiedae* have not been sequenced. With the advances in sequencing technologies, the RNA-Seq combined with SMRT method is being widely applied to analyze transcriptomes of various parasite species, with or without available reference genomes, which increases gene discovery, the accuracy of alternative splice detection, gene structure characterization, and long noncoding RNA prediction [38–40]. In the present study, we combined long-read SMRT with short-read RNA-Seq sequencing to generate a complete transcriptome of *E. stiedae*. A total of 456,652 CCS reads were obtained, yielding 12,582 full-length, non-redundant transcripts (N50 = 1972 bp; Table 2). Such data are much longer than those of other *Eimeria* parasites obtained using RNA-Seq [41] and therefore could contribute to a comprehensive understanding of developmental biology of *E. stiedae* and its interactions with hosts at the molecular level. Moreover, 5538 *E. stiedae*-specific transcripts might be also helpful to accurately characterize the *E. stiedae* transcriptome landscape and perform functional explorations of species-specific genes. For example, some of these transcripts were differentially expressed across the *E. stiedae* developmental stages and assigned to the “Transport and catabolism” pathway in the “Metabolism” category (Fig. 6d), suggesting their possible transport and catabolism activities in disposing accumulation of metabolites in different *E. stiedae* stages.

On basis of the analysis of DEGs between Ou, Os, Mz, and Gc, we revealed the transcriptomic dynamics across the *E. stiedae* life cycle. The genes that were upregulated during development from Ou to Os included those involved in membrane components, membrane complex, pellicle, and microtubules, consistent with observations in other *Eimeria* spp. [34, 41], which is in agreement with a conserved biological process involving sporozoite propagation taking over this stage transition. Following Os ingestion, the parasite triggers the transition from Os to Mz and Gc and is reflected by a significant upregulation of genes involved in cell components including organelle, cytoplasm, and ribonucleoprotein complex, and in biological processes including localization, single-organism process, regulation of biological process, reproductive process, signaling, and response to stimulus, which together guarantee its host invasion and merozoite reproduction as well as subsequent gametogony and parasite–host immuno-interactions. Interestingly, numerous genes related to the peptide biosynthetic process and peptide metabolic process is also accompanied with such transition, implying an active protein expression occurring in the Mz and Gc stages. This might be an adaptive strategy for preparing proteins to assist aforementioned cell invasion, parasite proliferation, and/or division processes. Compared to life-cycle development from Ou to Os and Mz, limited DEGs were found between Mz and Gc and most genes related to gametophyte development and gamete generation, supporting gametogony predominating in the transition. Taken together, these molecular clues revealed the hitherto unknown molecular developmental process of *E. stiedae*.

Based on current knowledge and our understanding of immune responses against eimerian parasites in animals, excretory/secretory (ES) proteins are regarded as immunomodulatory or immunogenic effectors of these parasites [42–44]. In our analysis of gene co-expressions of Mz and Gc, we identified numerous genes that are probably involved in host immune regulations. Among them were significant representatives such as secreted/excreted peptides and their inhibitors, including neutrophil inhibitory factors, lectins, TTL proteins, and peptidase inhibitors. These proteins within the *E. stiedae* secretome are predicted to direct or suppress immune responses and are close homologs of *N*-acteylglycosaminyltransferase and leucyl aminopeptidase ES-62 of nematodes [45]. ES-62 is known to inhibit B-cell, T-cell, and mast cell proliferation and responses, inducing a Th2 response through inhibition of IL-12p70 production by dendritic cells, and promotes alternative activation of host macrophages via the inhibition of Toll-like receptor (TLR) signaling [46, 47]. Other molecules of *E. stiedae* predicted to be immunomodulatory include homologs of cysteine proteases (CPBs) and serpins [48–50]. Moreover, certain *E. stiedae* ES proteins were predicted to be involved in immune evasion; for instance, lectins could mask parasite antigens by mimicking host molecules [51–53]. In spite of the similarities among eimerian parasite–host systems, based on the nature and extent of molecules identified here, we hypothesized that the host immune responses against the parasitic stages of *E. stiedae* might be distinct from those associated with other *Eimeria* spp., such as *E. maxima* [54]. Further experimental evidence is required to test this hypothesis. Nevertheless, the present findings indicated that *E. stiedae* has a substantial arsenal of ES proteins that are likely to be involved in modulating, evading, and/or blocking immune responses in the host.

In addition, we found that the genes encoding the apicomplexan AP2 (AP2) family of DNA-binding proteins, *Eimeria*-specific SAGs, CMGC kinase, DEAD/DEAH box helicase domain-containing protein, and proliferation-associated protein 2G4 (PA2G4) predominated at Mz4 of *E. stiedae* (Additional file 14: Table S11). Early studies showed that the AP2 DNA-binding protein family is a major class of transcriptional regulators and can prompt the transition from asexual to sexual replication of many apicomplexan parasites [55–57], such as *Cryptosporidium parvum* [58], *Theileria annulata* [59], and *Toxoplasma gondii* [60]. Within the genus *Eimeria*, the number of genes containing AP2 domains differs among species, and 21 *Eimeria*-specific AP2 groups and 22 other groups are shared by *Eimeria* and other coccidia, as well as five pan-apicomplexan clusters [61]. In the present study, we detected 20 genes containing AP2 domains in *E. stiedae* Mz4. Regarding the SAGs, *E. stiedae* expressed 75 SAGs, which could be further divided into sagA, sagB, and sagC subfamilies. It was obvious that the expression levels of the sagA group peaked at each stage, while sagB peaked in merozoites, which was similar to results reported for *E. tenella* [61]. About a half of the SAGs (*n* = 36) were upregulated in Mz4, which could be associated with the developmental fates of Mz. In *E. necatrix*, Mz2 are liberated from the second-generation meronts and passed to the cecum, where they penetrate the epithelial cells and develop into third-generation meronts. Thus, low expression of SAGs from Mz2 to Mz3 might be beneficial for parasites to escape from host immune responses [57]. However, considering no other generation of meronts was included here, we are not sure whether a similar case would also occur in mammal-infecting *Eimeria* species. The CMGC proteins are considered to play key roles in signaling pathways related to development and cell homeostasis, and DEAD/DEAH box proteins act in RNA metabolism, including transcription, splicing, translation, and decay [57, 62, 63]. Both appeared in the Mz4 and Gc stages of *E. stiedae* (Fig. 7; Additional file 15: Table S12), suggesting that they have roles related to life-cycle transitions from the free-living to parasitic phases. Additionally, PA2G4 associated with cell growth, differentiation, and apoptosis was upregulated in both Mz4 and Gc in the present study. A recent study indicated that PA2G4 negatively regulated intestinal inflammation by mediating the Akt signaling pathway [64]. Similarly, the upregulation of PA2G4 in both Mz4 and Gc of *E. stiedae*, to some extent, suggested that it might be capable of escaping immune responses by attenuating inflammatory damage of its host, as previously described [57].

## Conclusions

In summary, we carried out an analysis of the *E. stiedae* transcriptome with a combination of the PacBio SMRT long-read and Illumina short-read sequencing approaches. A total of 12,582 full-length transcripts were obtained, including 5538 novel transcripts. Comparative transcriptome analysis depicted the developmental dynamics of *E. stiedae* at the molecular level and also uncovered numerous genes that are related to parasite–host immune interactions. These findings provided important information for the characterization of the *E. stiedae* transcriptome and valuable molecular resources for veterinary *Eimeria* species and other apicomplexan parasites.

## Supplementary Information

**Additional file 1: Table S1.** RNA-Seq sample information.

**Additional file 2: Table S2.** RNA-Seq mapped read counts by full-length transcripts obtained from SMRT sequencing.

**Additional file 3: Table S3.** Normalized FPKM (fragments per kilobase per million mapped reads) read counts by full-length transcripts obtained from SMRT sequencing.

**Additional file 4: Table S4.** The quantitative real-time reverse transcription PCR (qRT-PCR) primers used for RNA-Seq validation.

**Additional file 5: Table S5.** Summary of the Iso-Seq transcriptome for *Eimeria stiedae*.

**Additional file 6: Table S6.** Assembly completeness as assessed using BUSCO in the coccidia lineage.

**Additional file 7: Figure S1.** Homologous species distribution of the *Eimeria stiedae* BLAST hits in the ToxoDB database.

**Additional file 8: Table S7.** The details of the functional annotation using public databases.

**Additional file 9: Table S8.** GO annotation of the *Eimeria stiedae* full-length transcripts.

**Additional file 10: Table S9.** KEGG annotation of the *Eimeria stiedae* full-length transcripts.

**Additional file 11: Table S10.** DEGs in at least one pairwise comparison of different life stages of *Eimeria stiedae*, with *p* values and GO hits.

**Additional file 12: Figure S2.** Venn diagram of differentially expressed genes (DEGs) in different life-stage comparisons of *Eimeria stiedae*.

**Additional file 13: Figure S3.** Volcano plots of differentially expressed genes (DEGs) between different life stages of *Eimeria stiedae*. The red and blue dots indicate significantly upregulated and downregulated genes, respectively (padj < 0.05), and the black dots represent the genes whose difference in expression level did not reach significance (padj > 0.05).

**Additional file 14: Table S11.** GO enrichment of up- and downregulated genes between developmental stages in *Eimeria stiedae*.

**Additional file 15: Table S12.** GO enrichment of nine clusters associated into five different groups based on the extent of shared genes among them. Note: Each group was characterized by having a high level of normalized transcription in one of developmental stages of *Eimeria stiedae*.

**Additional file 16: Figure S4.** Sample clustering based on Euclidian distance matrix according to the expression levels of all transcripts used for weighted gene coexpression network analysis (WGCNA).

**Additional file 17: Figure S5.** Visualizing the gene network using a heatmap plot. The heatmap depicts the Topological Overlap Matrix (TOM) among all transcripts in the analysis. Light color represents low overlap and red color represents higher overlap.

**Additional file 18: Figure S6.** Three upregulated modules (MEgreen, MEblue, and MEbrown) which were significantly correlated with Ou, Os, and Gc, respectively (see the black boxes in Fig. 8b).

## Data Availability

The comprehensive transcriptome data sets of *E. stiedae* have been deposited with links to BioProject number PRJNA698271, with accession numbers SRR13590427–SRR13590439 for RNA-Seq and SRR13684648 for PacBio SMRT data.

## Abbreviations

DEGs: Differentially expressed genes
MJ: Moving junction
MICs: Microneme proteins
AMAs: Apical membrane antigens
RONs: Rhoptry neck proteins
SAGs: Surface antigens
Ou: Unsporulated oocysts
Os: Sporulated oocysts
Mz4: Fourth-generation merozoites
Gc: Gametocytes
p.i.: Post-infection
CCS: Circular consensus sequence
FLNC: Full-length non-chimeric reads
ICEC: Iterative clustering and error correction
LoRDEC: Long-Read De Bruijn Graph Error Correction
NCBI: National Center for Biotechnology Information
Nr: Non-redundant proteins
COG: Cluster of Orthologous Groups of proteins
KEGG: Kyoto Encyclopedia of Genes and Genomes
GO: Gene Ontology
FDR: False discovery rate
RPKM: Reads per kilobase of transcript per million mapped reads
WGCNA: Weighted gene co-expression network analysis
TOM: Topological Overlap Matrix
GS: Gene significance
qRT-PCR: Quantitative reverse transcriptase polymerase chain reaction
GAP: Glideosome-associated proteins
STPKs: Serine/threonine protein kinases
ME: Module eigengene
ES: Excretory/secretory
TLR: Toll-like receptor
CPBs: Cysteine proteases
PA2G4: Proliferation-associated protein 2G4
FPKM: Fragments per kilobase per million mapped reads
