# Supplementary material for: Global transcriptome landscape of the rabbit protozoan parasite Eimeria stiedae
Source: Parasit Vectors. 2021 Jun 7;14:308. doi: 10.1186/s13071-021-04811-5 (PMC8186055; doi:10.1186/s13071-021-04811-5)
Supplement: Supplementary file 1 — Additional file 1: Table S1. RNA-Seq sample information. [file 13071_2021_4811_MOESM1_ESM.pdf]

Additional file 1: Table S1. RNA-seq sample information

Sample codes	Sequencing abbreviation (3 replicates)	Manuscript abbreviation	Description	Microscopic image
201918#1	W1		Unsporulated oocysts	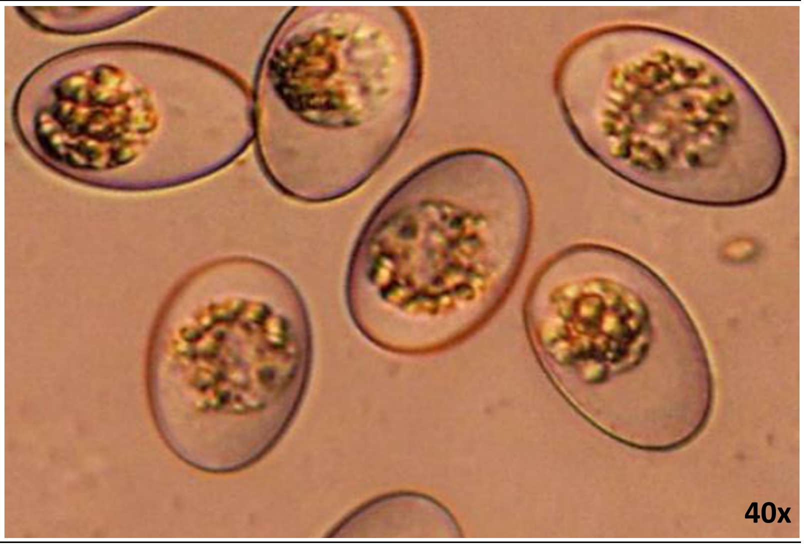
201918#2	W2	Ou	Unsporulated oocysts	
201918#3	W3		Unsporulated oocysts	
201918#4	B1		Sporulated oocysts	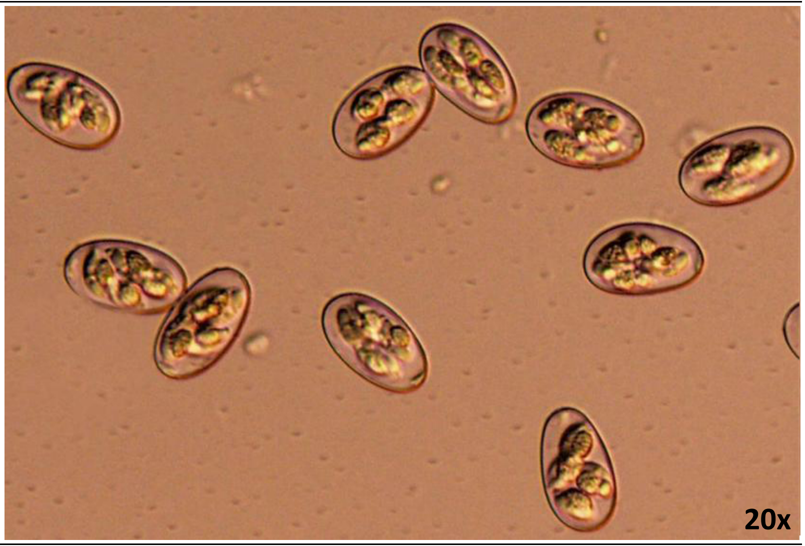
201918#5	B2	Os	Sporulated oocysts	
201918#6	B3		Sporulated oocysts	
201929#1	M1		Fourth generation merozoites	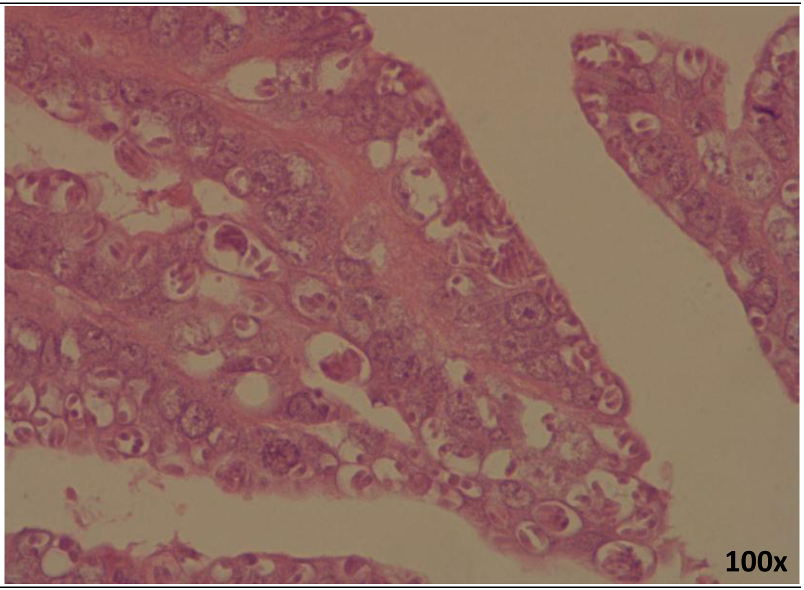
201929#2	M2	Mz4	Fourth generation merozoites	
201929#3	M3		Fourth generation merozoites	
201936#1	G1		Gametocytes	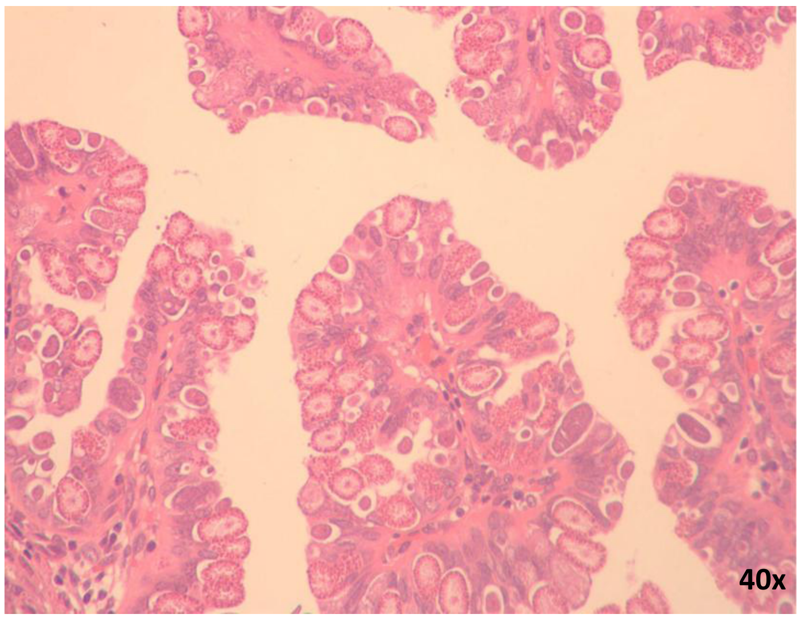
201936#2	G2	Gc	Gametocytes	
201936#3	G3		Gametocytes	
