# Supplementary figures and images for: Global transcriptome landscape of the rabbit protozoan parasite Eimeria stiedae
Source: Parasit Vectors. 2021 Jun 7;14:308. doi: 10.1186/s13071-021-04811-5 (PMC8186055; doi:10.1186/s13071-021-04811-5)

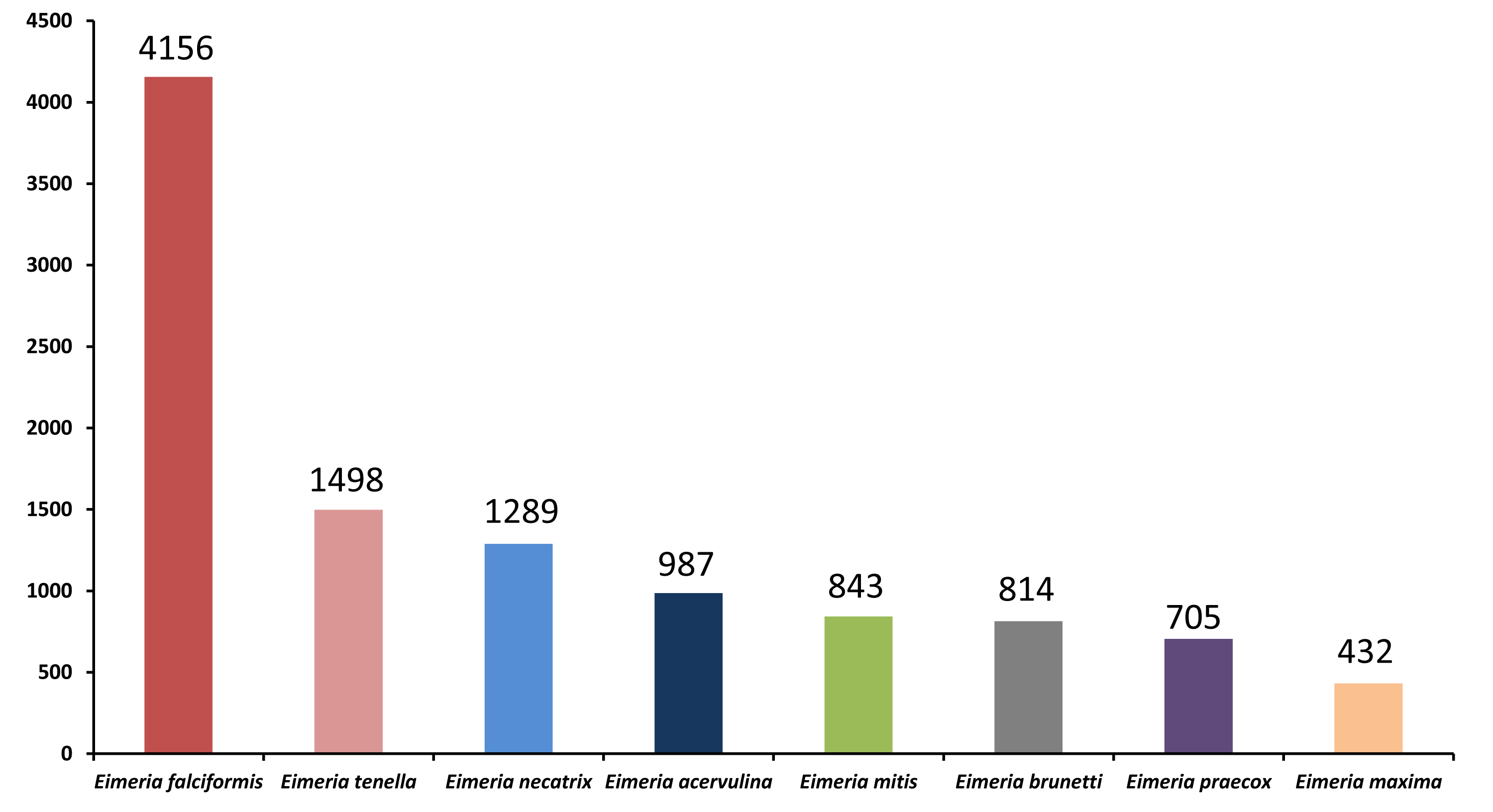

Supplement: Supplementary file 7 — Additional file 7: Figure S1. Homologous species distribution of the Eimeria stiedae BLAST hits in the ToxoDB database. [file 13071_2021_4811_MOESM7_ESM.tif]

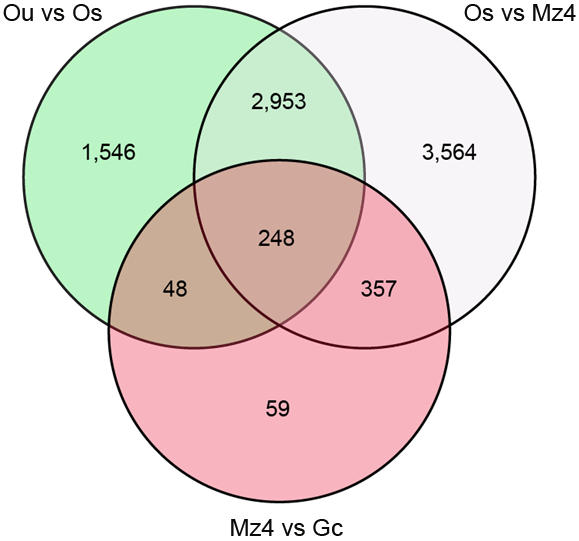

Supplement: Supplementary file 12 — Additional file 12: Figure S2. Venn diagram of differentially expressed genes (DEGs) in different life-stage comparisons of Eimeria stiedae. [file 13071_2021_4811_MOESM12_ESM.tif]

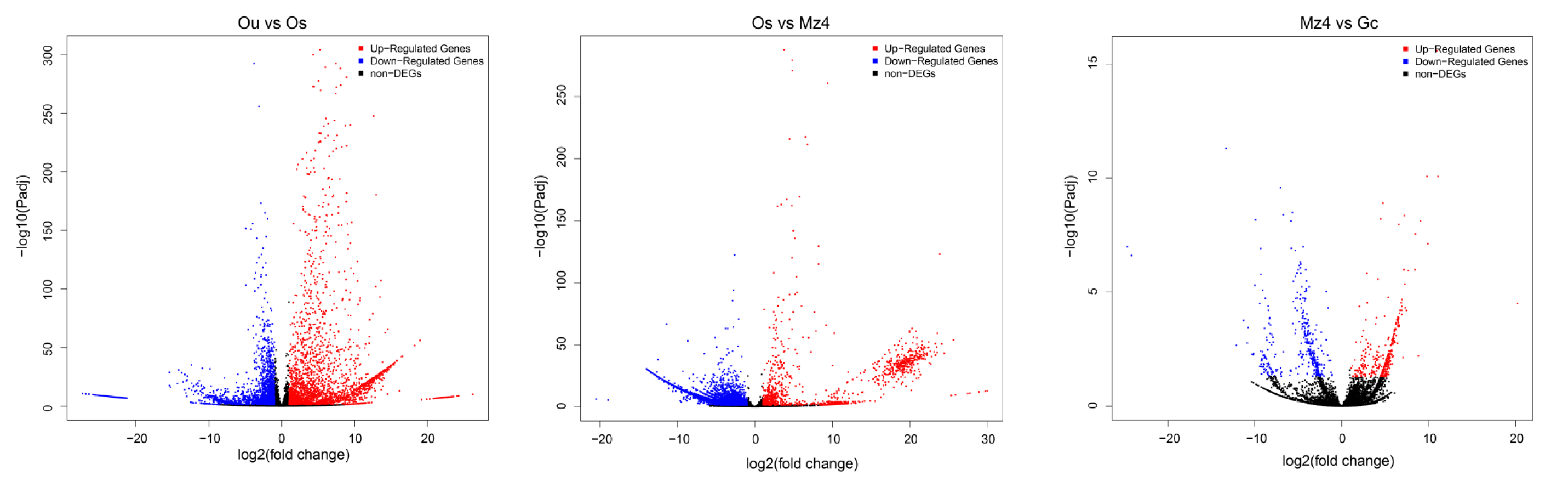

Supplement: Supplementary file 13 — Additional file 13: Figure S3. Volcano plots of differentially expressed genes (DEGs) between different life stages of Eimeria stiedae. The red and blue dots indicate significantly upregulated and downregulated genes, respectively (padj < 0.05), and the black dots represent the genes whose difference in expression level did not reach significance (padj > 0.05). [file 13071_2021_4811_MOESM13_ESM.tif]

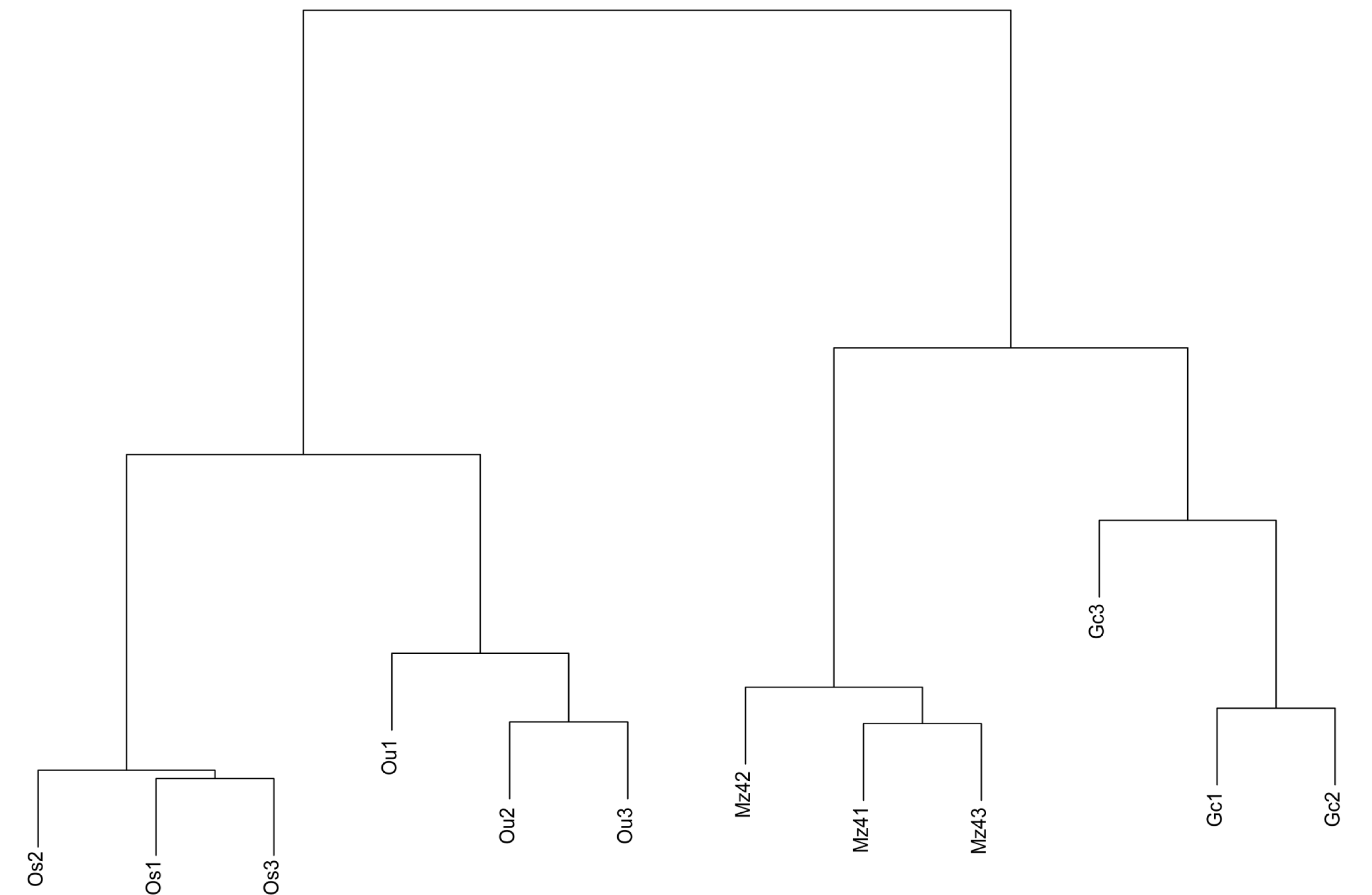

Supplement: Supplementary file 16 — Additional file 16: Figure S4. Sample clustering based on Euclidian distance matrix according to the expression levels of all transcripts used for weighted gene coexpression network analysis (WGCNA). [file 13071_2021_4811_MOESM16_ESM.tif]

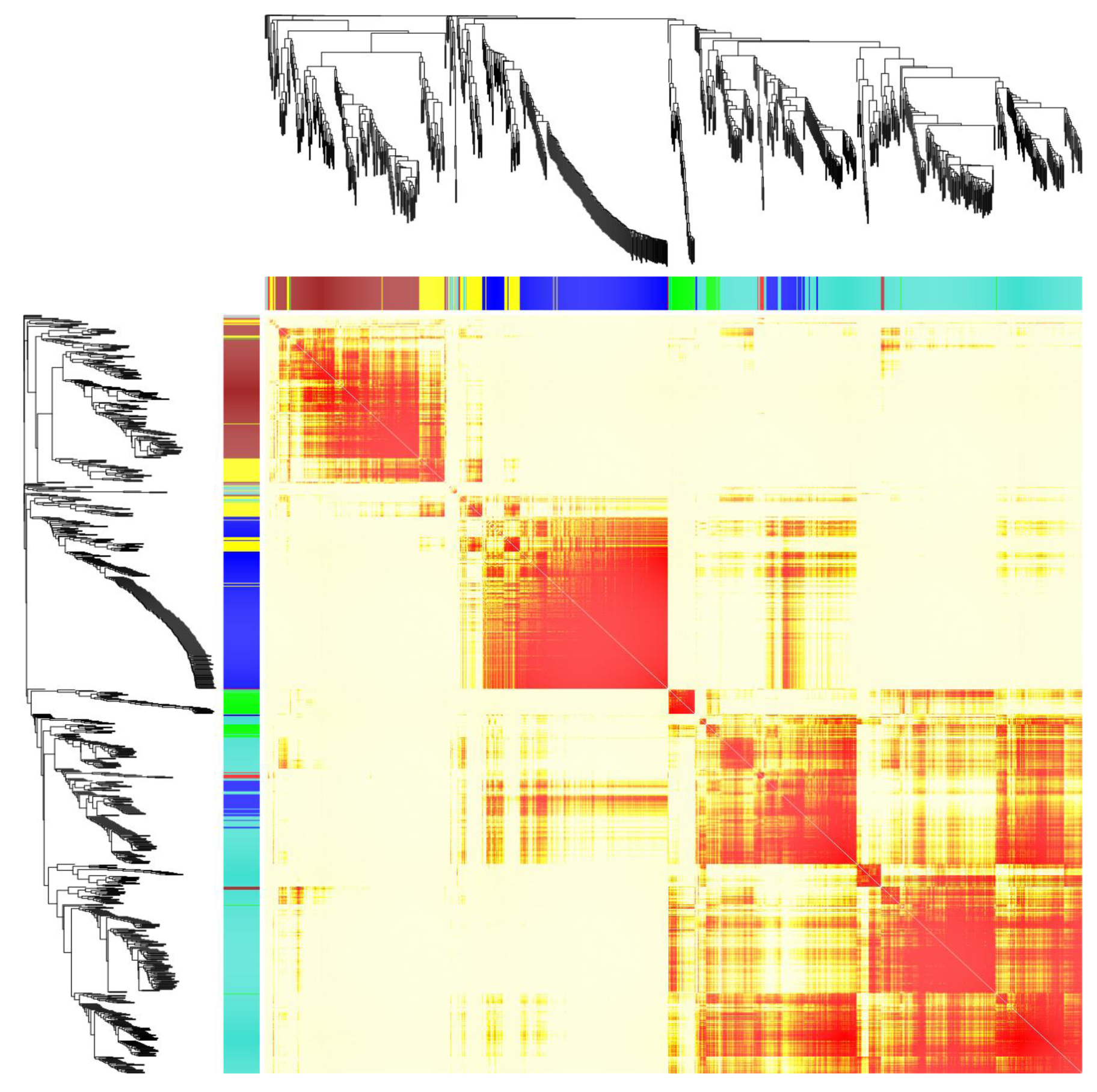

Supplement: Supplementary file 17 — Additional file 17: Figure S5. Visualizing the gene network using a heatmap plot. The heatmap depicts the Topological Overlap Matrix (TOM) among all transcripts in the analysis. Light color represents low overlap and red color represents higher overlap. [file 13071_2021_4811_MOESM17_ESM.tif]

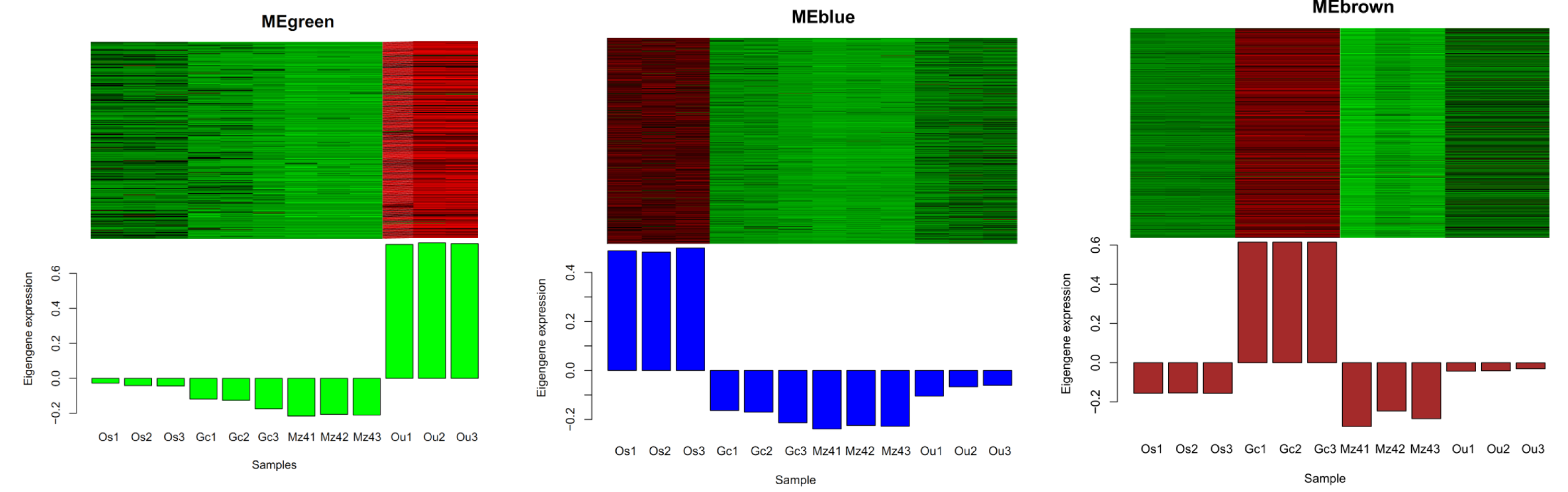

Supplement: Supplementary file 18 — Additional file 18: Figure S6. Three upregulated modules (MEgreen, MEblue, and MEbrown) which were significantly correlated with Ou, Os, and Gc, respectively (see the black boxes in Fig. 8b). [file 13071_2021_4811_MOESM18_ESM.tif]
